# Ethical issues posed by cluster randomized trials in health research

**DOI:** 10.1186/1745-6215-12-100

**Published:** 2011-04-20

**Authors:** Charles Weijer, Jeremy M Grimshaw, Monica Taljaard, Ariella Binik, Robert Boruch, Jamie C Brehaut, Allan Donner, Martin P Eccles, Antonio Gallo, Andrew D McRae, Raphael Saginur, Merrick Zwarenstein

**Affiliations:** 1 Rotman Institute of Philosophy, Department of Philosophy, University of Western Ontario, London, ON, N6A 5B8, Canada; 2 Department of Medicine, University of Western Ontario, 339 Windermere Road, London, ON, N6A 5A5, Canada; 3 Department of Epidemiology and Biostatistics, University of Western Ontario, London, ON, N6A 5C1, Canada; 4 Ottawa Hospital Research Institute, Clinical Epidemiology Program, Civic Campus, 1053 Carling Avenue, Ottawa, ON, K1Y 4E9, Canada; 5 Department of Medicine, Faculty of Medicine, University of Ottawa, ON, K1H 8L6, Canada; 6 Department of Epidemiology and Community Medicine, University of Ottawa, ON, K1H 8M5, Canada; 7 Graduate School of Education and Statistics Department, Wharton School, University of Pennsylvania, 3700 Walnut Street, Philadelphia, PA, 19104, USA; 8 Robarts Clinical Trials, Robarts Research Institute, London, ON, N6A 5K8, Canada; 9 Institute of Health and Society, Newcastle University, Baddiley-Clark Building, Richardson Road, Newcastle upon Tyne, NE2 4AX, UK; 10 Schulich School of Medicine and Dentistry, University of Western Ontario, London, ON, N6A 5C1, Canada; 11 Division of Emergency Medicine, University of Calgary, Foothills Medical Centre, 1403 29th Street NW, Calgary, AB, T2N 2T9, Canada; 12 Department of Medicine, University of Ottawa and Ottawa Hospital, Ottawa Hospital Research Institute, 1053 Carling Avenue, Ottawa, ON, K1Y 4E9, Canada; 13 Centre for Health Services Sciences, Sunnybrook Health Sciences Centre, 2075 Bayview Avenue, Toronto ON, M4N 3M5, Canada

## Abstract

The cluster randomized trial (CRT) is used increasingly in knowledge translation research, quality improvement research, community based intervention studies, public health research, and research in developing countries. However, cluster trials raise difficult ethical issues that challenge researchers, research ethics committees, regulators, and sponsors as they seek to fulfill responsibly their respective roles. Our project will provide a systematic analysis of the ethics of cluster trials. Here we have outlined a series of six areas of inquiry that must be addressed if the cluster trial is to be set on a firm ethical foundation:

1. Who is a research subject?

2. From whom, how, and when must informed consent be obtained?

3. Does clinical equipoise apply to CRTs?

4. How do we determine if the benefits outweigh the risks of CRTs?

5. How ought vulnerable groups be protected in CRTs?

6. Who are gatekeepers and what are their responsibilities?

Subsequent papers in this series will address each of these areas, clarifying the ethical issues at stake and, where possible, arguing for a preferred solution. Our hope is that these papers will serve as the basis for the creation of international ethical guidelines for the design and conduct of cluster randomized trials.

## Introduction

The cluster randomized trial is an increasingly important method in health research. Cluster trials randomize intact social units, such as households, primary care practices, hospital wards, classrooms, neighborhoods and entire communities, to differing intervention arms. Research interventions in cluster trials may be directed at the entire cluster or at individual cluster members. Compared with an individually randomized trial with the same number of individuals, cluster trials are inefficient and have less statistical power [1]. This is a result of the fact that the responses of individuals within a cluster tend to be more similar than the responses of individuals in differing clusters [1]. Accordingly, the use of a cluster randomized design must be carefully justified. The cluster randomized design is used appropriately in a number of circumstances.

First, the nature of the intervention may require that it be administered at the cluster level. For instance, the Community Intervention Trial for Smoking Cessation (COMMIT) used mass education -- a cluster-level intervention -- to target entire communities in an attempt to reduce smoking rates. The trial promoted smoking cessation through a wide range of influences including public education, health care workers, and employers and with such broad interventions, randomization of individuals would have been impossible [2].

Second, interventions may involve training or education of health professionals with the aim of improving patient care. For example, Lewin and colleagues examined the impact on patient outcomes of a cluster-level training programme for health workers caring for tuberculosis patients in South Africa [3]. The study targeted primary care clinics in Cape Town that had tuberculosis treatment completion rates of less than 70%. In the intervention arm of the trial, nurse clinicians underwent an 18 hour in-service training program that focused on patient centered care and quality improvement. Study outcomes compared patient treatment completion and patient cure rates before and after the study intervention. Here again, an intervention targeting a provider who treats many patients often makes patient-specific randomization unfeasible or impossible.

Third, the investigators may desire to reduce the effect of treatment contamination. For instance, Kennedy and colleagues studied the effect of patient-centered educational materials -- an individual-level intervention -- on patient knowledge, anxiety, and quality of life [4]. Patients in the study were on long term follow-up for ulcerative colitis. As patients attending the same hospital clinic frequently interact with one another, the study randomized clusters of patients attending the same clinic to receive the educational materials or no intervention to avoid treatment contamination.

Fourth, investigators may wish to study both individual and group effects of an intervention. For example, vaccine researchers have employed cluster randomized trials to quantify both the direct and indirect effects of vaccination [5]. A vaccine administered to individuals within a community may directly protect an individual from infection by inducing protective antibodies or indirectly by virtue of the fact the person is surrounded by people who have developed protective antibodies to the disease (so-called "herd immunity"). A cluster randomized trial allows researchers to measure the protective effect of the vaccine both among those who are vaccinated and develop antibodies and in the community at large.

The literature exploring the design, analysis, and reporting of cluster randomized trials is expanding rapidly [6,7]. But cluster trials raise difficult ethical issues that have not been addressed adequately. A Canadian Institutes of Health Research funded project seeks to study ethical issues in health-related cluster randomized trials systematically to inform the development of international guidelines. As described elsewhere, the project involves three major components [8]. First, it seeks to document current practice through a systematic review of cluster trials, in-depth interviews with cluster randomization trialists, a survey of research ethics committees, focus group discussions, and in-depth interviews with trial participants and gatekeepers. Second, it aims to analyze comprehensively the ethical issues posed by cluster trials in a series of papers. Third, and finally, the project will convene an expert panel to develop guidelines for the ethical conduct and review of cluster trials.

This article introduces a series of papers from the second part of the larger project that explore ethical issues in health-related cluster randomized trials. In this paper, we explain the importance of ethical issues in cluster trials, review contemporary principles of research ethics, and define a series of ethical issues posed by cluster trials. Each of these issues is addressed in detail in a subsequent paper in the series.

## Importance of the problem

While there is a small but growing literature on the subject, the ethical issues raised by cluster randomized trials require further analysis. As a result, researchers currently lack authoritative guidance to help them design and conduct cluster trials according to the highest ethical standards. Research ethics committees and regulators have no single international standard to guide their review of cluster trials. Predictably, the lack of authoritative guidance has resulted in uncertainty and markedly different interpretations as to permissible practices in cluster trials. Consider the experience with two knowledge translation studies, the NEXUS trial conducted in the UK and the Keystone study in the United States.

In the NEXUS trial, Eccles and colleagues used a 2 × 2 factorial, cluster randomized design to study the effect of two interventions on general practitioners' use of radiographs [9]. In the study, 244 primary care practices in England and Scotland were randomly allocated to no intervention, audit and feedback, educational messages, or both in an attempt to reduce general practitioner requests for lumbar spine and knee radiographs in accord with UK Royal College of Radiologists' guidelines. Audit and feedback reports were shared with practices at baseline and six months and compared the number of requests for radiographs within the practice with all other practices in the previous six months. Educational messages were attached to reports of radiographs ordered during the 12 month intervention period. The outcome measure, using data routinely collected by radiology departments, was the number of each kind of radiograph request per 1000 patients registered with each practice. The trial concluded that educational messages reduced radiography referral requests by 20%, but found that audit and feedback had no impact on referral requests.

The NEXUS trial was approved by the West Midlands Multi-site Research Ethics Committee. Although the study interventions targeted general practitioners, informed consent was not obtained from them. Elsewhere, the study authors explain that

"we successfully argued that the trial interventions were the equivalent of low risk service developments and that the requirement to seek consent from all potential healthcare professionals may make the project unfeasible or bias our assessment of the study outcome. As a result, we informed all general practitioners within the study areas that there was an ongoing trial but did not explicitly seek their consent. When the interventions were rolled out, we received fewer than five complaints from over 1,000 general practitioners involved in the study" [10].

Furthermore, although the study sought to change the management of patients presenting with knee and lower back pain, informed consent was not sought from patients treated in the general practices participating in the study. The study authors argued that patients indirectly affected by the study intervention could not be identified at the time of randomization and it would be difficult or impossible to respect patient refusals. "If a patient decided that they [*sic*] did not want to receive care influenced by the intervention, how can the general practitioner minimize the influence of the intervention for the individual patient" [10]? The NEXUS trial is regarded as a model knowledge translation study and has been cited numerous times in the literature.

Compare the NEXUS trial with recent experience by the Michigan Health and Hospital Association Keystone Intensive Care Unit study (hereafter, the "Keystone Study"). While the Keystone Study was not a cluster randomized trial (it lacked randomization and a concurrent control group), it involved the administration of a knowledge translation intervention to health professionals and observed patient outcomes. The Keystone Study is described as a prospective cohort study involving 103 intensive care units that sought to reduce the rate of bloodstream infections resulting from central venous catheters [11]. A complex intervention targeted health professionals' use of procedures known to reduce catheter-related infections. The intervention included education of healthcare providers, the creation of a central line cart with needed supplies, a checklist to ensure adherence with procedures, stopping providers if they were not adhering to procedures, and routine discussion of catheter removal. Data on the number of catheter-days and catheter-related infections were collected and aggregated into three-month periods at baseline, during the intervention period, and for up to 18 months of follow up. The study results were impressive. Catheter-related bloodstream infection dropped from 2.7 infections per 1000 catheter-days at baseline to 0 three months after the intervention and remained low for the duration of follow up. If widely implemented, the complex intervention could cut catheter-related infection rates by half [12].

Like the NEXUS trial, the Keystone study was approved by a single research ethics committee, in this case, the institutional review board at Johns Hopkins University. The institutional review board determined that the study was exempt from federal regulations on the basis that it involved "the collection or study of...[information] recorded by the investigator in such a manner that subjects cannot be identified" [13]. Accordingly, the institutional review board did not require researchers to obtain the informed consent of health care providers or patients in the study. Shortly after the publication of the Keystone study, the U.S. Office for Human Research Protections (OHRP) -- the government agency that oversees institutional review boards in the U.S. -- received an anonymous complaint that the Keystone study had not been conducted in accord with federal regulations [14]. The OHRP investigation found that the institutional review board at Johns Hopkins University erred in considering the study exempt from federal regulations, institutional review board review should have been conducted at all participating sites, and that informed consent should have been obtained from both the health professionals and the patients (or their surrogates) in the study. As a result, the Keystone study was suspended and the continuing collection of follow-up data was halted.

The conflicting experiences of the NEXUS trial and the Keystone study reveal deep disagreements on basic ethical issues. For instance: When is a study human subjects research? Who is a research subject? And from whom, how, and when must informed consent be obtained? Until these questions can be answered, uncertainty will remain. According to Kass and colleagues, the "moral hazard of this uncertainty is that fewer formal patient safety studies may be undertaken, resulting in a slowdown in progress..." [15].

## A standard view of research ethics

We begin our exploration of ethical issues posed by cluster randomized trials by considering a standard view of research ethics. Our current understanding of the ethics of clinical research is largely based on individually randomized trials. Typically, in these trials the research subject is simultaneously the unit of randomization, the unit of experimentation, and the unit of observation. Commonly, a patient is allocated randomly to receive one of two differing treatment regimens and data documenting the patient's response to the treatment received are recorded. Because such studies target individuals, the ethics of clinical research is focused on the protection of the liberty and welfare interests of individual research subjects. Liberty interests include a right of freedom from interference without informed consent and a right of confidentiality. Welfare interests include the interest to receive treatment consistent with competent medical care, and the interest not to be exposed to undue risk for the benefit of third parties.

According to Levine, "[t]he term 'research' refers to a class of activities designed to develop or contribute to generalizable knowledge" [16]. Research ethics may be viewed as governed by four ethical principles: respect for persons; beneficence; justice; and respect for communities [16,17]. The principle of respect for persons requires that researchers take seriously the choices of autonomous people, that is, people who can responsibly make their own decisions. Importantly, people lacking autonomy, such as young children or adults with advanced dementia, are entitled to protection. The principle of respect for persons is the source of the moral rules of informed consent and confidentiality (table 1). The researcher is generally obligated to obtain agreement from a research subject (or his or her surrogate decision maker) for study participation. In order for informed consent to be valid, the research subject must have the cognitive capacity to make the choice, be so situated as to choose freely, have adequate information, and understand what is at stake in the decision. Informed consent may not be required when it cannot practicably be obtained and study participation poses only minimal risk. Researchers must also take necessary steps to protect the confidentiality of the research subject's health information.

**Table 1:** Ethical principles and rules for the conduct of clinical research. (Adapted from [17])

Moral Principle	Moral Rule
Respect for persons	Obtain the informed consent of prospective research subjects (or their surrogate decision makers).
	Protect the confidentiality of private information.

Beneficence	Therapeutic procedures must satisfy clinical equipoise.
	Risks of non-therapeutic procedures must be (1) minimized and (2) reasonable in relation to knowledge to be gained.

Justice	Subject selection procedures must be fair.
	Compensate subjects harmed as a result of research participation.

Respect for communities	Respect communal values, and protect and empower social institutions.
	Where applicable, abide by the decisions of legitimate communal authority.

The principle of beneficence obliges researchers not to harm needlessly and, where possible, to promote the good of research subjects. Clinical research often contains a mixture of study procedures, some offering reasonable prospect of benefit to research subjects (therapeutic procedures), while others are administered solely to answer the scientific question (nontherapeutic procedures). According to a systematic approach to the ethical analysis of benefits and harms in research called component analysis, therapeutic and nontherapeutic procedures must be considered separately [18]. Therapeutic procedures, such as drugs or surgical procedures, are justified if they satisfy clinical equipoise, meaning they must be comparable with competent medical care. In other words, there must be a state of honest, professional disagreement in the community of expert practitioners as to the preferred treatment [19]. Non-therapeutic procedures, such as additional blood tests or questionnaires that are not clinically indicated, do not offer the prospect of benefit to research subjects. Non-therapeutic procedures are acceptable if the risks associated with them are minimized consistent with sound scientific design, and reasonable in relation to the knowledge to be gained. When the study involves a vulnerable population, such as children or incapable adults, the risks posed by nontherapeutic procedures must not exceed a minor increase above minimal risk. According to component analysis, one may only conclude that the benefits and harms of a study are acceptable when the moral rules for both therapeutic and non-therapeutic procedures are satisfied (table 1).

The principle of justice may be defined as the ethical obligation to distribute the benefits and burdens of research fairly. Researchers have an obligation to ensure that study procedures for the selection of research subjects are equitable. Researchers must neither exploit the vulnerable, nor exclude without good reason those who stand to benefit from study participation. In order for proposed eligibility criteria to be evaluated, each criterion must be accompanied by a clear justification in the study protocol [20]. The inclusion of a vulnerable group (such as children, incapable adults, prisoners, or pregnant women) requires a clear justification. Further, in so far as is possible and practicable, the study population ought to mirror the target clinical population. The historical exclusion -- in certain cases -- of children, women, and racial minorities from the benefits of research has led to a variety of contemporary initiatives to promote their inclusion in clinical research [21,22]. The principle of justice also requires that provisions be in place to compensate research subjects who are harmed as a result of research participation [23].

A novel ethical principle of respect for communities has been proposed [24]. The principle of respect for communities implies that investigators have an obligation to respect communal values, protect and empower social institutions, and, where applicable, abide by the decisions of legitimate communal authorities. There is much support for the principle. First, the community (or communities) to which we belong is an important source of values and self-understanding. Second, a community consists of social structures that are essential to the well-being of its members. Third, the principle acknowledges that some communities already exercise power legitimately to make binding decisions on behalf of members, for instance in the collection of taxes or the setting of speed limits on roads. Practically, the researcher-community relationship ought to be viewed as a partnership in which community partners are involved from study design through publication [25].

## Ethical issues posed by cluster trials

Cluster randomized trials only partly fit within the current paradigm of research ethics. They pose difficult ethical issues for two basic reasons. First, cluster trials involve groups rather than (merely) individuals, and our understanding of the moral status of groups is incomplete. As a result, the answers to pivotal ethical questions, such as who may speak on behalf of a particular group and on what authority they may do so, are unclear. Second, in cluster trials the units of randomization, experimentation, and observation may differ, meaning, for instance, that the group that receives the experimental intervention may not be the same as the group from which data are collected (e.g., in the NEXUS trial, the intervention was directed at primary care physicians and the outcome was the frequency of patient x-rays). The implications for the ethics of trials of experimental interventions with (solely) indirect effects on patients and others is currently not well understood. Based on review of the literature, interviews with cluster randomization trialists, the practical experiences of team members, and group discussion, members of the CIHR funded project identified six ethical areas of inquiry related to cluster trials in need of further exploration and analysis. Below we introduce each of these ethical issues. Subsequent papers in the series will address in detail each of these domains of inquiry.

### 1. Who is a research subject?

To determine whether the ethical principles and regulations governing research apply, one must first conclude that a study is human subjects research and then identify the research subjects. Indeed, much of the debate on the Keystone study focused on whether the study was in fact human subjects research [14,26-28]. For instance, Baily argued that the Keystone study is not human subjects research:

"The project was not designed to use ICU patients as human subjects to test a new, possibly risky method of preventing infections; rather, it was designed to promote clinicians' use of procedures already known to be safe and effective for the purpose. Each hospital engaged in a classic quality-improvement activity in which team members worked together to introduce best practices and make them routine, with quantitative feedback on outcomes being intrinsic to the process. Such activities should not require IRB review." [26].

But neither novelty nor risk is at the core of what constitutes human subjects research. Rather, recalling Levine's definition of research above, research is a systematic intervention designed to produce generalizable knowledge. Miller and Emanuel argue that "the project was...[human subjects research] since it prospectively implemented a protocol of infection control interventions and tested hypotheses regarding its effectiveness. Publication of the study results suggests that a goal was to produce generalizable results" [14].

While the line between quality improvement activities and human subjects research can be very difficult to draw [29], we believe the distinction is rarely an issue for cluster randomized trials. The difficult issue for cluster trials is to establish who counts as a research subject. The question is of considerable importance, as only research subjects properly fall under the aegis of research ethics committees and protections such as informed consent. Knowledge translation cluster trials commonly intervene on healthcare providers but measure outcomes on patients. Are the healthcare providers research subjects in this case? What about the patients? Other cluster trials, such as the COMMIT study, involve community level interventions and collect data on a subset of community members. Are just those who were sampled for data research subjects, or are all members of the community research subjects?

U.S. regulations define a research subject as a "living individual about whom an investigator...conducting research obtains (1) Data through intervention or interaction with the individual, or (2) Identifiable private information". It goes on to define 'interventions' as "both physical procedures by which data are gathered...and manipulations of the subject or the subject's environment that are performed for research purposes" [30]. With respect to knowledge translation trials, when healthcare workers are the target of the study intervention it might be argued that they are, as a result, research subjects. But in such studies, if patients are only indirectly impacted by the study intervention and if no identifiable private information is collected, should we consider them research subjects? (This, we take it, might be a better way of understanding Baily's point about the Keystone study.) What about community members in a cluster trial in which the intervention is applied at the level of the community? The answer depends on how we understand the phrase "manipulations of...the subject's environment" [31]. To identify who is a research subject in cluster trials, we need a clear understanding of what sorts of environmental manipulations properly invoke the protective apparatus of research ethics and regulation. Each of these issues is explored in a subsequent article in the series.

### 2. From whom, how, and when must informed consent be obtained?

The ethical principle of respect for persons generally requires that researchers obtain the informed consent of research subjects. In the cluster trials literature it is commonly claimed that the need for informed consent from individuals depends on whether the study intervention is delivered at the level of the cluster or the individual [32,33]. With a cluster-level intervention, individual refusal of informed consent may be, in effect, rendered meaningless. If an individual within a cluster refuses study participation he or she will, in many cases, be unable to avoid exposure to the study intervention and this undermines the very purpose of consent [33]. As Edwards and colleagues put it, in such studies, "the autonomy principle is lost except insofar as the individual has any democratic choice of who the guardian is and some right to consultation by the guardian" [32]. When the intervention targets individual research subjects, generally informed consent can and should be obtained. In these cases, "it is only trial entry that takes place without individual consent, as the individual treatments offered can be declined or accepted by each participant. This resembles a conventional trial where consultation over consent implies that available alternatives are offered and that these always include routine care" [32].

While this approach seems broadly correct, further work will need to justify in terms familiar to research ethics committees and regulators why individual consent may not be required in cluster trials when the intervention targets the cluster. We see two possible justifications. First, ethical and regulatory requirements for informed consent apply only to research subjects. If it turns out that, for instance, patients or community members who are only indirectly impacted by the study intervention are not research subjects, then informed consent is *ipso facto *not required. Second, requirements for informed consent may be waived if four conditions obtain: the research poses no more than minimal risk; the rights and welfare of subjects are not adversely affected; the research could not be carried out practicably otherwise; and, when appropriate, subjects will be debriefed [34]. The applicability of these criteria to cluster trials requires further analysis to provide researchers and research ethics committees with practical guidance. When does a cluster trial pose only minimal risk to subjects? When does a waiver of consent not adversely affect the rights and welfare of research subjects? How rigorously are we to understand the requirement that the research could not *practicably *be carried out? Must subjects be debriefed and, if so, how should this be done?

When must informed consent be obtained from healthcare workers in cluster trials? There are at least three dimensions of this issue that require further consideration. First, when the study intervention targets an entire hospital or primary care practice it may be difficult for a healthcare worker who refuses consent to avoid the study intervention. Second, health care workers are commonly believed to have an obligation to engage in quality improvement. Third, as Hutton and colleagues point out, "if a health care professional chooses not to participate in a study, they [*sic*] are in effect denying their patients the potential benefits of participation. Healthcare providers ought to do the best for their patients..." [10].

When the study intervention is administered at the individual level, it is generally agreed that the informed consent of the research subject must be obtained [33]. But when a cluster trial involves a behavioral intervention, the informed consent process may lead to treatment contamination [35,36]. Edwards and colleagues explain that

"[i]nforming the controls fully about the experimental arm(s) is likely to produce the very effect that randomizing by cluster was designed to avoid -- that is, prompting controls to adopt the treatment(s) under investigation. One option is to withhold information about the novel treatment from controls, on the grounds that they are getting conventional care and are therefore in the same position as people outside the experiment" [32].

But can information about the details of the study intervention be withheld from research subjects in the control arm consistent with the principle of respect for persons?

Finally, Klar and Donner raise a difficult question regarding the timing of informed consent that requires further exploration. To illustrate their concern they point to two studies examining the impact of vitamin A administration on early childhood mortality. In the first study, the unit of randomization was the household and informed consent was obtained from study participants prior to randomization [37]. In the second study, the unit of randomization was the community and informed consent was only obtained after randomization [38]. The authors worry that

"[t]he relative absence of ethical guidelines for cluster randomized trials appears to have created a research environment in which the choice of randomization unit may determine whether informed consent is deemed necessary before random assignment...It seems questionable, on both an ethical level and a methodological level, whether the unit of randomization should play such a critical role in deciding whether informed consent is required [before randomization]" [39].

A subsequent paper in the series examines each of these questions in detail.

### 3. Does clinical equipoise apply to CRTs?

The ethical principle of beneficence obliges researchers to not harm needlessly and, where possible, to promote the good of research subjects. The application of beneficence to cluster trials raises two broad questions.

First, do researchers have an ethical obligation to research subjects in the control arm to provide more than usual care? The question arises out of the belief that, while subjects in the experimental arm may benefit as a result of study participation, those in the control arm are exposed to risks and burdens without the prospect of such benefit. Glanz and colleagues state:

"Meeting [ethical] requirements...is particularly challenging when individuals or communities are assigned to control or comparison groups that do not receive the intervention hypothesized to be most effective. The control subjects may be burdened disproportionately by data collection requirements without receiving the benefit of services or resources" [36].

According to Klar and Donner, "some investigators have attempted to ensure that these individuals can still benefit from participation by offering a minimal level of intervention or, alternatively, by offering all individuals the intervention by the technique of delaying its intervention in the control group" [39]. While intuitively appealing, these approaches require further reflection. If denying research subjects in the control arm access to the hoped for benefits of the experimental intervention is ethically impermissible, then why is it permitted to give them only "minimal" benefits or to delay their access to these benefits?

Second, as data accumulate in a cluster trial, is there an obligation to modify or stop the study if one of the interventions appears unsafe or unexpectedly effective? For a variety of reasons, data monitoring committees are not commonly used in cluster randomized trials. When data monitoring committees are employed, they require clear guidance as to their ethical obligations. Glanz and colleagues have argued that concerns of safety or unexpected efficacy may require a data monitoring committee to modify or stop a study prematurely [36]. They point out that "interim analysis could show a clear improvement in psychological or medical outcomes associated with an intervention. It would then be reasonable to offer the more effective strategy to all communities or participants" [36]. It is well recognized that early differences between interventions may be the result of chance or bias rather than a true intervention effect. How much evidence of a "clear improvement" ought there be before a data monitoring committee recommends that a study ought to be modified or stopped?

In the literature on individually randomized trials, the concept of clinical equipoise helpfully frames questions regarding researcher obligations to subjects in the control group and when data monitoring committees ought to recommend modifying or stopping a clinical trial. As described above, clinical equipoise permits a trial to be started when there exists a state of honest, professional disagreement in the community of expert practitioners as to the preferred treatment [19]. By implication, a trial ought to be stopped when the moral warrant for its conduct no longer obtains [40]. It is unclear, however, whether clinical equipoise can be applied to cluster trials. The concept is commonly understood as emerging from the fiduciary relationship between physician-researcher and patient-subject [41]. Cluster trials may involve neither physician-researchers nor patient-subjects. For instance, in both the NEXUS trial and the Keystone study, the targets of the study intervention were health care workers themselves. In the COMMIT study, the targets of the study intervention were communities and community members. If clinical equipoise is to be used to address issues posed by cluster trials, a moral foundation relevant to cluster trials will have to be articulated for it. The applicability of clinical equipoise to CRTs is considered in detail in a subsequent paper in the series.

### 4. How do we determine if the benefits outweigh the risks of CRTs?

The principle of beneficence requires that the benefits of study participation stand in reasonable relation to its risks. Numerous publications describe the variability in review from one research ethics committee to the next. For instance, Hearnshaw documents wide discrepancies in requirements for ethics review and time to approval in 11 European countries for a study involving an information pamphlet and questionnaire for elderly patients and their physicians [42]. While part of the variation in ethics review is a result of regulatory differences among countries, the lack of a structured approach to the ethical analysis of risk is thought to be an important contributing factor. Described in detail above, component analysis provides research ethics committees with a systematic approach to the ethical analysis of benefits and harms in research [18]. The applicability of component analysis to cluster randomized trials is, unfortunately, unclear. If component analysis is to be applied to cluster trials, a number of conceptual hurdles will first have to be cleared.

Does the distinction between therapeutic and nontherapeutic procedures hold in cluster trials? The first step in component analysis is the demarcation of therapeutic and nontherapeutic procedures. The distinction between therapeutic and nontherapeutic procedures is generally unproblematic in cluster trials with an individual level intervention. These individual level interventions commonly are drug, surgical, or behavioral interventions that aim to benefit research subjects, and, thus, they are straightforwardly therapeutic interventions. The difficulty is posed by cluster trials involving a cluster level intervention. Public health trials commonly involve a cluster level intervention designed to improve the health of a community and its members. For instance, the COMMIT trial employed a multimedia campaign to increase quit rates in heavy smokers and reduce the prevalence of smoking in the community. Ought we to understand these interventions as therapeutic? Even more difficult to classify are complex interventions that aim to modify healthcare worker behavior in knowledge translation trials. The NEXUS trial used audit and feedback and educational messages to attempt to reduce physician orders for needless radiographs. Should we classify these procedures as therapeutic or nontherapeutic interventions?

The second step in component analysis is to ask whether therapeutic procedures meet the ethical standard of clinical equipoise [18]. Question #3 above considers in detail the applicability of clinical equipoise to cluster trials. The third step in component analysis is to ask whether the risks of nontherapeutic procedures are minimized consistent with sound scientific design, stand in reasonable relation to the knowledge to be gained, and, if the study involves a vulnerable population, pose no more than a minor increase above minimal risk [18]. The applicability of each of these standards to cluster trials deserves exploration. Of particular interest is the meaning of minimal risk in the context of a cluster trial. Minimal risk is commonly defined as the risks of daily life of a healthy person [43]. When cluster trials target households, neighborhoods, or communities, it is unclear whether an individualistic understanding of minimal risk remains appropriate. Might minimal risk refer to the quotidian risks faced by clusters rather than individuals? What impact would such an understanding have on the review of cluster trials? A subsequent paper in the series analyzes these questions in detail.

### 5. How ought vulnerable groups be protected in CRTs?

The principle of justice requires that vulnerable groups in research both be protected adequately and not unduly denied access to research benefits. Vulnerable groups are commonly understood to include pregnant women, prisoners, children, and incompetent adults, and cluster trials have studied all of these groups. Althabe and colleagues describe a cluster trial of a multifaceted behavioral intervention to improve obstetrical care in Argentina and Uruguay [44]. Hickman and colleagues randomized specialist drug clinics and prisons to test whether the use of dried blood spots to test for hepatitis C would increase uptake of diagnostic testing by injection drug users [45]. Kipping and colleagues describe a pilot cluster trial in which schools with children 9 and 10 years of age were randomized to receive an obesity prevention intervention or no intervention [46]. De Smet and colleagues randomized 13 intensive care units in the Netherlands to receive digestive tract decontamination with oral and intravenous antibiotics, digestive tract decontamination with oral antibiotics only, and usual care in an attempt to reduce 28-day patient mortality [47].

A variety of additional protections apply when clinical research involves a vulnerable group. The inclusion of the vulnerable group in research must be required to answer the study hypothesis; a vulnerable group cannot be used merely as a population of convenience. When prospective research subjects are incapable of providing informed consent, a surrogate decision maker must provide consent on their behalf. Finally, the risks of nontherapeutic procedures must not exceed a minor increase above minimal risk. Cluster trials, particularly those involving interventions applied at the level of the cluster, may further restrict the ability of vulnerable groups (or their surrogate decision makers) to choose research participation freely. Does this imply that greater protections for vulnerable groups in cluster trials are required? How might one meaningfully enhance protections without impeding research that may benefit the health of vulnerable groups?

Research conducted in developing countries raises a host of ethical issues [17]. Consider Bolton and colleagues' description of the first cluster randomized trial of psychotherapy in sub-Saharan Africa [48]. Depression is a common and serious health problem in sub-Saharan Africa, with a prevalence estimated at 21% [48]. Unfortunately, few treatments are available for those suffering from depression in impoverished countries. Antidepressant drugs are too expensive and psychotherapy, developed for use in industrialized countries, has not been tested for efficacy. In the trial, 30 villages in rural Uganda were randomized to receive psychotherapy or usual care. Study subjects were identified with the help of community leaders, healers, and other knowledgeable persons and, after they provided verbal informed consent, the diagnosis of depression was confirmed with a culturally appropriate questionnaire. In villages allocated to the intervention arm, subjects received group-based interpersonal psychotherapy for 90 minutes each week for 16 weeks. In control villages, research subjects were free to seek out whatever interventions they wished. Symptoms were again assessed after the intervention period. The study intervention proved highly effective in treating depression: after the intervention, 6.5% of subjects in the intervention group met the criteria for severe depression, compared with 54.7% of subjects in the control group. Upon completion of the study, group psychotherapy was made available to the control communities.

The trial of interpersonal psychotherapy for depression illustrates both the potential and challenges of cluster trials in developing countries. Researchers conducting cluster trials face ethical issues that flow from cultural differences and disparities in access to health care between host and sponsor countries. What ethical standards, including those for informed consent, ought to apply: those of the host or sponsor country? What treatment should research subjects in the control arm receive? Clinical equipoise requires patients enrolled in a trial not be exposed to treatment known to be inferior to treatments available in clinical practice. But, one might ask, available where? In developed countries, standard treatment for major depression includes antidepressant drugs and psychotherapy. Must subjects in the control arm receive the best, proven therapy, even if it is locally unavailable? In communities with substandard access to healthcare, do researchers have an obligation to provide research subjects with treatment for medical conditions not related to the study condition? What obligations do researchers and study sponsors have to research subjects and host communities after completion of the study? Do they have a moral obligation to provide participating communities with access to the study intervention (if it proves effective), and, if so, for how long? These important justice issues are explored in a subsequent paper in the series.

### 6. Who are gatekeepers and what are their responsibilities?

There is a growing consensus in the research ethics literature that researchers have obligations to communities participating in research. The ethical principle of respect for communities flows from the recognition that the community has moral worth and, as a result, researchers have a duty to protect and promote its interests [24]. The community-researcher relationship has been described as a partnership, in which community consultation and negotiated agreement are key features [25]. When a community has a legitimate political authority empowered to speak on behalf of its members, researchers may additionally be required to seek community consent to research participation. Importantly, community consent does not supplant the requirement for individual informed consent to study participation. While protections for communities in research may straightforwardly apply to cluster trials in which the unit of randomization is the community, their applicability across the scope of cluster trials is uncertain. Cluster trials randomize diverse groups that are not communities -- households, primary care practices, hospital wards, classrooms, and neighborhoods -- and whose moral status is not well characterized. In the cluster trials literature, the gatekeeper has emerged as a key player in protecting the interests of these diverse groups and their members [32,33,49]. However, a variety of questions regarding the role, function, and authority of gatekeepers have yet to be explored adequately.

Who are gatekeepers? When cluster trials involve an intervention that is administered at the cluster level, difficulties in obtaining meaningful individual informed consent have led to the practice of using gatekeepers [49], guardians [32], and cluster representation mechanisms [33] to protect group and individual interests. Edwards and colleagues define a gatekeeper as "an agent...who has the power to 'deliver' [a] cluster," and who acts as an advocate on behalf of cluster interests [32]. Hutton, defines gatekeepers as "people in either political or administrative positions who are able to give consent for those within a cluster to be randomized" and whose consent may occur on multiple "levels" [49]. Current descriptions of gatekeepers, however, do not give a clear account of who can act as a gatekeeper when there are no clear administrative or political structures in place. The diversity of groups studied in cluster trials poses a challenge to how we identify gatekeepers, and how group characteristics influence who may serve as representatives.

What are the functions of gatekeepers? Gatekeepers are described as being able to "deliver" [32] or "give consent for" [49] a cluster. The U.K. Medical Research Council guidelines describe the role of a gatekeeper as "analogous...to that of individuals for individual decisions" and says the gatekeeper must act "in the interests of the cluster/individuals in the cluster" [33]. Further, the gatekeeper must document that he or she "considers the cluster's participation in the trial to be in the interests of the cluster as a whole/in the interests of each member of the cluster (as appropriate...)" [33]. The potential for conflict among the various sets of interests protected by the gatekeeper requires careful examination. Acknowledging that community or cluster and individual interests are separable and may be conflicting [24,50], how should a gatekeeper balance individual and cluster interests if they conflict? Gatekeepers, who may be in administrative positions (e.g., practice managers, hospital chief executive officers), will also have to balance cluster and institutional interests and consider the impact of the research on the organization for which they are responsible.

What are the sources of a gatekeeper's authority? One of the outcomes of the debate on community consent is the recognition of the importance of the issue of authority [24]. Only communities that possess a legitimate political authority empowered to speak on behalf of its members may provide community consent. The issue of authority for the variety of functions ascribed to gatekeepers requires careful scrutiny. When does a gatekeeper possess the authority to consent on behalf of the cluster? When individual consent cannot be obtained, does a gatekeeper have the authority to consent on behalf of the individuals in the cluster? A subsequent paper in the series critically appraises the role, function, and authority of gatekeepers in CRTs.

## Conclusion

The cluster randomized trial is used increasingly in knowledge translation research, quality improvement research, community based intervention studies, public health research, and research in developing countries. However, cluster trials raise difficult ethical issues that challenge researchers, research ethics committees, regulators, and sponsors as they seek to fulfill responsibly their respective roles. Our project will provide a systematic analysis of the ethics of cluster trials. Here we have outlined a series of six areas of inquiry that must be addressed if the cluster trial is to be set on a firm ethical foundation. Subsequent papers in this series will address each of these areas, clarifying the ethical issues at stake and, where possible, arguing for a preferred solution. Our hope is that these papers will serve as the basis for the creation of international ethical guidelines for the design and conduct of cluster randomized trials.

## Note

We have created a Wiki webpage to facilitate an open discussion about the ideas expressed in this and other papers published in the series on ethical issues in CRTs. Please enter your thoughts and comments at http://crtethics.wikispaces.com.

## Abbreviations

COMMIT: Community Intervention Trial for Smoking Cessation; CRT: Cluster randomized trial; ICU: Intensive care unit; Keystone Study: Michigan Health and Hospital Association Keystone Intensive Care Unit Study; NEXUS: North East X-Ray Utilization Study; OHRP: U.S. Office for Human Research Protections; RCT: Randomized controlled trial;

## Competing interests

AB, JCB, AG, ADM, RS, MT, CW: None declared

RB, AD, MPE, JMG, and MZ have all submitted cluster trial protocols to ethics committees and had difficulty explaining to them the differences between cluster randomized trials and individual patient randomized trials.

## Authors' contributions

CW, MT, and JMG contributed to the conception and design of the manuscript.

CW wrote the initial draft and led writing of subsequent versions.

All authors commented on sequential drafts and approved the final version.
